# Relapse prediction using wearable data through convolutional autoencoders and clustering for patients with psychotic disorders

**DOI:** 10.1038/s41598-025-03856-1

**Published:** 2025-05-29

**Authors:** April Yujie Yan, Traci Jenelle Speed, Casey Overby Taylor

**Affiliations:** 1 Department of Biomedical Engineering, Johns Hopkins School of Medicine, Baltimore, MD USA; 2 Institute for Computational Medicine, Johns Hopkins Whiting School of Engineering, Baltimore, MD USA; 3 Department of Psychiatry and Behavioral Sciences, Johns Hopkins University School of Medicine, Baltimore, MD USA; 4 Division of General Internal Medicine, Department of Medicine, Johns Hopkins University School of Medicine, Baltimore, MD USA

**Keywords:** Psychotic disorders, Relapse prediction, Unsupervised machine learning, Digital mental health, Digital phenotyping, Psychiatric disorders, Predictive markers

## Abstract

Relapse of psychotic disorders occurs commonly even after appropriate treatment. Digital phenotyping becomes essential to achieve remote monitoring for mental conditions. We applied a personalized approach using neural-network-based anomaly detection and clustering to predict relapse for patients with psychotic disorders. We used a dataset provided by e-Prevention grand challenge (SPGC), containing physiological signals for 10 patients monitored over 2.5 years (relapse events: 560 vs. non-relapse events: 2139). We created 2-dimensional multivariate time-series profiles containing activity and heart rate variability metrics, extracted latent features via convolutional autoencoders, and identified relapse clusters. Our model showed promising results compared to the 1^st^ place of SPGC (area under precision-recall curve = 0.711 vs. 0.651, area under receiver operating curve = 0.633 vs. 0.647, harmonic mean = 0.672 vs. 0.649) and added to existing evidence of data collected during sleep being more informative in detecting relapse. Our study demonstrates the potential of unsupervised learning in identifying abnormal behavioral changes in patients with psychotic disorders using objective measures derived from granular, long-term biosignals collected by unobstructive wearables. It contributes to the first step towards determining relapse-related biomarkers that could improve predictions and enable timely interventions to enhance patients’ quality of life.

## Introduction

Psychotic disorders, a group of serious mental disorders across a range of psychiatric illnesses that are characterized by the presence of symptoms including delusions, hallucinations, disorganized thinking, abnormal motor behavior, and negative symptoms (e.g., diminished emotional expression), which can significantly affect daily functioning and quality of life^1^. Affecting approximately 4.6 per 1,000 people globally, these disorders often follow a relapsing–remitting course^2^. Even though numerous studies have explored the causes of psychotic disorders, effective biomarkers to detect relapse remain unestablished due to insufficient understanding of the pathophysiology of psychosis^3^. Thus, biomarkers for timely diagnosis and intervention of relapse are a prominent area in psychiatry to detect clinically significant changes in disease states^4–6^. Relapse, broadly defined as the return or worsening of symptoms of partial recovery, occurs commonly even after initiation of antipsychotic medications^3,7,8^. Early identification of worsening symptoms contributes significantly to the prevention of catastrophic effects that relapses often have on patients’ lives^9–11^. Moreover, since relapse develops over time, it would be reasonable to anticipate changes in biomarker signals preceding the onset of worsening symptoms^12–14^.

Digital phenotyping, defined as “the quantification of individual-level human phenotype in situ,” has emerged as an essential tool for personalized detection of mental conditions, such as relapse, depressive episodes, and stress^15–18^. Today, digital devices are driving the development of relapse prediction systems, accelerating a paradigm shift in mental health care by enabling dynamic, long-term modeling and patient-specific care^19^. The N-of-1 study approach^15^, where individual-level models are trained separately for each person, is no longer futuristic; it offers significant potential for addressing population heterogeneity in digital mental health. A study^15^ based on two clinical trials (50 and 10 analyzed patients, respectively), consisting of active and passive data collected over 1 year, developed a personalized anomaly-detection framework to predict relapse in patients with major depressive disorders. Another study^16^ on predicting psychotic relapse using long-term mobile data (63 patients, 27 relapse events) proposed patient-specific models through clustering-based behavioral characterization. While the field is emerging, challenges remain for collecting complete, granular, and long-term data.

Significant efforts have been made to enable such data collection due to the need for high-quality, long-term, high-frequency data through digital devices (e.g., wearables and smartphones). Open-source platforms such as mindLAMP facilitate worldwide participation and collect both active (user responses via surveys) and passive (biosignals from wearable sensors) data for mental health research^20^. The U.S.-based All-of-Us initiative^21^ provides individual-level data from a large, diverse population—including socio-demographics, electronic health records, minute-level wearable signals monitored over years, and survey responses—to registered researchers. For instance, a study^22^ has identified a large cohort with thousands of patients consisting of up to 120-day wearable signals per patient to detect depression. Moreover, the e-prevention project released a public dataset with approximately 2,700 days of high-frequency wearable data from 10 patients with psychotic disorders, supporting studies that showed great potential for personalized prediction of relapse using highly granular, long-term biosignals^3,23–25^.

Many recent studies have explored relapse prediction in patients with psychotic disorders using individual, long-term wearable signals. A pilot study analyzed 15 patients with schizophrenia, with 5 experiencing a relapse^8^. Daily features were summarized from active and passive data collected by a smartphone application, Beiwe. A study leveraged the potential of supervised neural networks and achieved personalized relapse prediction based on a public dataset, CrossCheck, consisting of 63 patients (20 relapse patients)^26^. Another study based on CrossCheck (18 relapse patients) focused on unsupervised learning and passive data^27^. However, they employed daily or hourly features with less granularity than the e-prevention data.

Neural networks have emerged as a promising tool for detecting and identifying biomarkers derived from wearable biosensors to predict mental status changes^28^. The e-Prevention project made contributions to this direction by collecting physiological time-series data on patients with psychotic disorders (e.g., bipolar disorder with psychotic features and schizophrenia) and analyzing biomarkers (e.g., heart-rate variability (HRV) metrics) associated with relapses^3^. Several works utilized anomaly detection and examined autoencoders that reconstruct data from wearables to identify unusual patterns in biomarkers related to relapse^3,27,29,30^. Such unsupervised-learning, neural-network-based methods, and clustering algorithms were effective in detecting insomnia patterns and cardiovascular diseases wearable data^31,32^. Unsupervised learning showed more potential than supervised methods in extracting latent patterns for detecting anomalies.

**Fig. 1:**
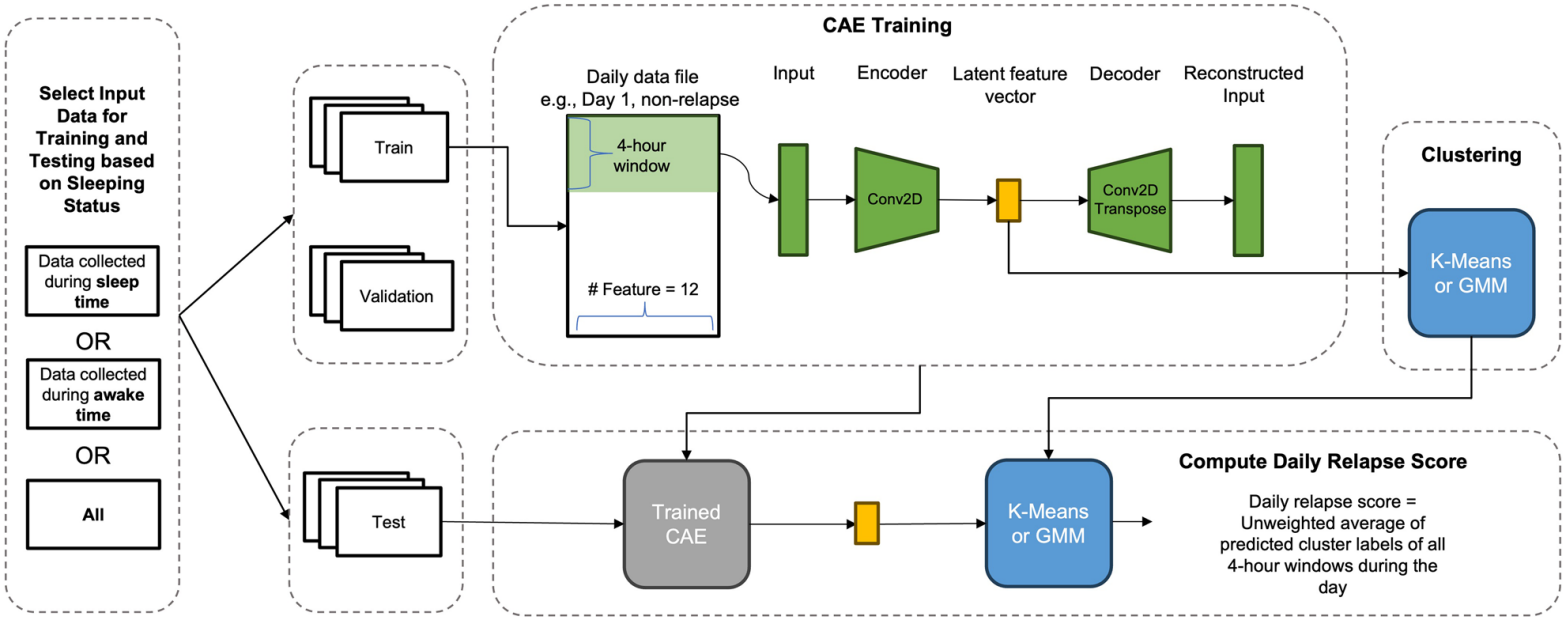
Overall workflow and CAE architecture. The workflow consists of four stages: (1) data selection based on sleep status (sleep/awake/all), (2) CAE training, (3) generate cluster labels regarding relapse status, and (4) computation of daily relapse scores. Each daily data file includes 5-min intervals across a day; we extract 4-h windows as 2D input profiles. The encoder applies a 2D convolutional filter (height = 11, width = 12) to learn latent feature vectors, while the decoder reconstructs input profiles via convolution transpose, minimizing MSE. The resulting latent features are clustered to identify relapse patterns. The trained CAE and best-performing clustering algorithm on validation data are used for test data, and daily relapse scores are computed from cluster labels. Model performance is evaluated using PR-AUC, ROC-AUC, and their harmonic mean.

**Fig. 2:**
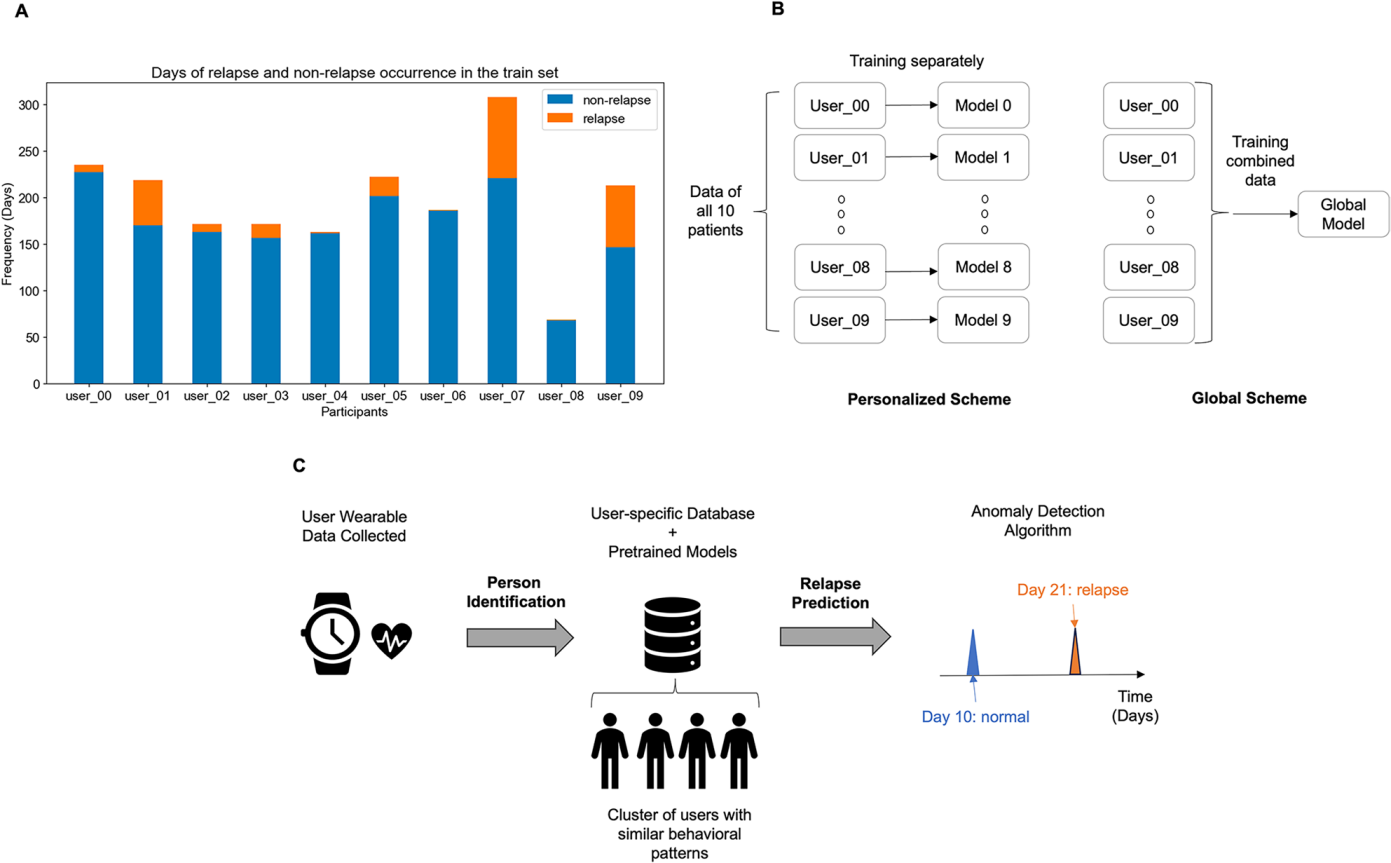
Demonstration of outcome imbalance and training schemes. (**A**) days of relapse and non-relapse occurrence for each patient; (**B**) data distribution and model training based on personalized vs. global scheme; (**C**) envisioned framework for personalized remote monitoring of mental conditions (e.g., relapse).

Despite the potential of applications of unsupervised, neural-network-based anomaly detection in wearable data and psychiatry, little effort has been made to cluster patterns of relapse in patients with psychotic disorders using objective measures derived from highly granular, long-term signals of physical and cardiac activities. In this work, we hypothesized that distinct clusters representing relapse and/or abnormal behaviors exist among patients with psychotic disorders. To test the hypothesis, we investigated an unsupervised anomaly-detection method by leveraging granular (e.g., minute-level), long-term wearable data of patients with psychotic disorders. Using data provided by ICASSP Signal Processing Grand Challenge (SPGC) 2023, we created 2-dimensional (2D) time-series profiles, containing 12 activity and HRV features, and predicted daily relapse events via 2D convolutional autoencoders (CAE) and clustering (Fig. 1). SPGC dataset adopted a working definition of relapse that encompasses both psychotic and non-psychotic symptom exacerbations, consistent with the broader clinical characterization of relapse in psychotic disorders. Given previous studies identifying the impact of sleep status on relapse detection in patients with psychotic disorders^25^, we conducted stratified analyses based on data collected during sleep vs. awake time (Fig. 1). We adopted the steps of an unsupervised anomaly-detection framework^33^ where autoencoders were only trained using non-relapse data and validation were based on both relapse and non-relapse data. We trained personalized models where CAE training and clustering were performed on a per patient basis (Fig. 2A and 2B). Essentially, as a proof-of-concept study, we provided more evidence showing the potential of an unsupervised learning and remote monitoring in personalized relapse prediction (Fig. 2C) and identified effective relapse-related objective measures derived from granular and long-term wearable data.

## Results

The SPGC dataset contains physiological signals of 10 patients with psychotic disorders, recorded over 2.5 years with a total of 2,699 days, consisting of 560 relapse days and 2139 non-relapse days (Table 1). Among the 10 patients, 2 have schizoaffective disorder, 4 have bipolar disorder I with psychotic features, 1 has brief psychotic disorder, 1 has schizophreniform disorder, and 2 have schizophrenia^3^. Other socio-demographics were not provided.

**Table 1:** Days of non-relapse and relapse occurrence in train/validation/test sets per patient.

Participant (# days in total)	Train set	Validation set		Test set	
	**Non-relapse** **(Days)**	**Non-relapse** **(Days)**	**Relapse** **(Days)**	**Non-relapse** **(Days)**	**Relapse** **(Days)**
User_00 (303)	227	27	8	31	10
User_01 (316)	170	17	49	23	57
User_02 (230)	163	19	9	26	13
User_03 (228)	157	18	15	21	17
User_04 (210)	162	19	2	23	4
User_05 (297)	202	25	20	28	22
User_06 (243)	186	23	2	27	5
User_07 (455)	221	24	87	29	94
User_08 (92)	68	4	2	14	4
User_09 (325)	147	16	66	22	74
Total (2699)	1703	192	260	244	300

We conducted training on a per-patient basis. For each patient, we trained their personalized CAE model using the train set consisting of only non-relapse data and the validation set consisting of both relapse and non-relapse data. Given the highly imbalanced nature of the data, with relapse days being rare, we adopted an anomaly-detection framework and trained the model exclusively on non-relapse days to better capture deviations indicative of relapse. We extracted a latent feature representation and used it for the subsequent clustering of relapse. Given previous investigations of the effect of sleep on relapse identification^25^, we designed experiments using various configurations. Specifically, we trained the CAE and performed clustering either separately on sleep and awake data or jointly on the combined data. Sleep periods were identified based on device-recorded sleep epochs, which provided individual-specific sleep start and end times for each day. For clarity, we refer to “sleep data” as physiological measurements collected during these personalized sleep windows, “awake data” as data collected outside these detected sleep periods, and “all data” as the combined data. In total, eight experimental setups were evaluated, as summarized in Table 2. For evaluation, we used each patient’s personalized CAE model to reconstruct their test data and extract latent features for clustering. Cluster labels (e.g., non-relapse or relapse) were then assigned to each 4-h window. To compute the daily relapse prediction score, we took the unweighted average of the predicted labels across all 4-h windows within each day. The overall performance metrics, aggregated across all ten patients, were reported in Table 3. When we trained CAE and performed clustering using sleep data (Experiment 7), we achieved the best performance with the area under the curve of precision-recall (PR-AUC) of 0.716, and area under the curve of precision-recall (ROC-AUC) of 0.633, and their harmonic mean of 0.672. To assess the separation between clusters, we computed the silhouette score, which was 0.18, indicating weak but interpretable separation in the latent feature space. Cluster structure was visualized using UMAP in Supplementary Figure 1, with colors indicating relapse and non-relapse groups. The results were comparable to the 1^st^ place from SPGC with the PR-AUC of 0.651, and ROC-AUC of 0.647, and harmonic mean of 0.649. We found that CAE trained using sleep data outperformed those trained using awake data. Specifically, CAE trained based on sleep data achieved a harmonic mean of 0.619 (Experiment 2) while training based on awake (Experiment 3) and all data (Experiment 1) achieved 0.580 and 0.536, respectively.

**Table 2:** Experiment setup.

Experiment	CAE training setup	Clustering setup
1	All data	Separate for sleep/awake data
2	Sleep data	Separate for sleep/awake data
3	Awake data	Separate for sleep/awake data
4	All data	All data
5	Sleep data	All data
6	Awake data	All data
7	Sleep data	Sleep only
8	Awake data	Awake only

**Table 3:** Evaluation results on test data of all 10 patients.

	**PR-AUC**	**ROC-AUC**	**Harmonic mean**
Experiment 1	0.582	0.496	0.536
Experiment 2	0.655	0.586	0.619
Experiment 3	0.631	0.537	0.580
Experiment 4	0.585	0.527	0.554
Experiment 5	0.594	0.589	0.592
Experiment 6	0.603	0.577	0.590
Experiment 7	0.716	0.633	0.672
Experiment 8	0.588	0.531	0.558
SPGC Baseline^34^	0.635	0.578	0.605
SPGC: PeRCeiVe^23^	0.651	0.647	0.649
SPGC: Emotion^24^	0.635	0.607	0.621
SPCG: SAILers^25^	0.636	0.584	0.605

Bold fonts represent the highest value of the column; baseline for PR-AUC (i.e., positive rate) = 0.551.

We demonstrated the robustness of the proposed algorithm considering patient variability. When training the algorithm using all data, sleep data, and awake data, we found that model performance across different patients was promising, with median harmonic means of 0.614, 0.670, and 0.561, respectively. Details can be found in Supplementary Tables 1–3.

**Fig 3.**
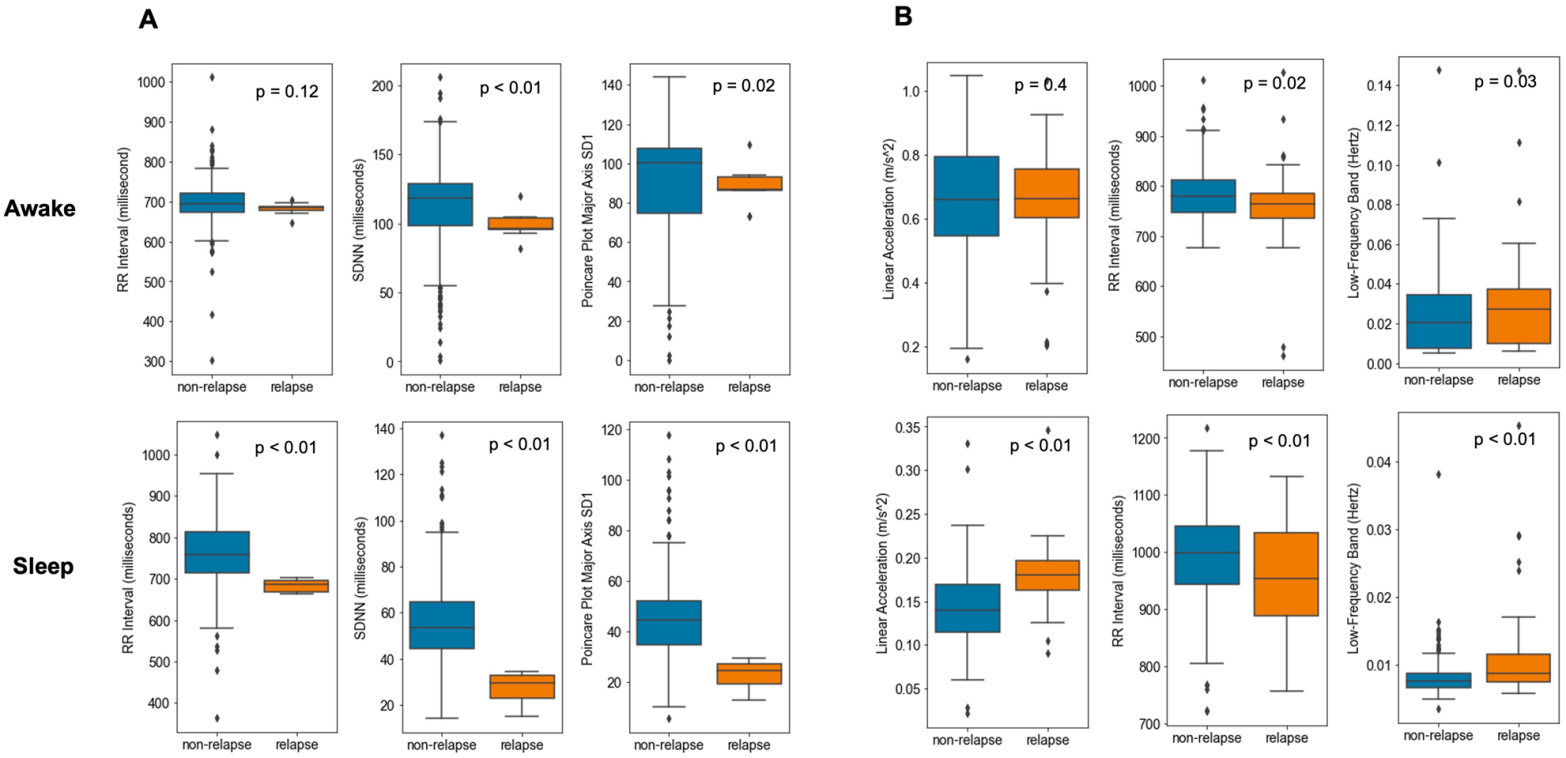
Comparison of feature distributions between non-relapse and relapse groups stratified by sleep status. (**A**) User_00 (from left to right): RR Interval (millisecond), SDNN (millisecond), Poincare Plot Major Axis SD1; (**B**) User_01 (from left to right): linear acceleration (meter/second^2^), RR-interval (millisecond), Low-Frequency Band of NN Interval (Hertz). Abbreviations: SD (standard deviation), RR (R-peak-to-R-peak), NN (normal-to-normal), SDNN (standard deviation of NN interval), LF (low-frequency). P-values were generated by Kolmogorov–Smirnov test and adjusted by Benjamini–Hochberg procedure.

We discovered that behavioral patterns related to relapse were more distinguishable during sleep for most patients through Kolmogorov–Smirnov (KS) test. Feature distribution plots (Fig. 3) demonstrated that behavioral patterns related to relapse were more separable during sleep than awake. For example, in User_00’s sleep data (Fig. 3A), the RR interval during non-relapse periods was significantly higher than during relapse (759 ± 89 vs. 683 ± 17 ms, p < 0.01). However, no significant difference was observed during awake periods (697 ± 60 vs. 681 ± 16 ms, p = 0.12). Similarly, for User_01 (Fig. 3B), linear acceleration during sleep was significantly lower in non-relapse periods compared to relapse (0.14 ± 0.04 vs. 0.18 ± 0.04 m per second^2^, p < 0.01), while no significant difference was observed during awake periods (0.66 ± 0.18 vs. 0.66 ± 0.17 m per second^2^, p = 0.40).

Moreover, the range and distribution of many features varied largely between sleep and awake data. For instance, in User_00’s data, the standard deviation of normal-to-normal intervals (SDNN) was much lower during sleep than during awake periods. Specifically, during sleep, SDNN was 56.5 ± 20.7 ms in non-relapse periods and 27.2 ± 7.6 ms in relapse periods. In contrast, during awake time, SDNN values were substantially higher: 110.4 ± 34.3 ms for non-relapse and 99.1 ± 10.4 ms for relapse (Fig. 3A). Similarly, User_01’s RR interval was notably higher during sleep than awake, with non-relapse and relapse values of 990 ± 90 ms and 956 ± 100 ms during sleep, compared to 785 ± 55 ms and 761 ± 79 ms during awake periods (Fig. 3B). Additional feature-level comparisons are provided in Supplementary Table4. Ignoring these differences led to suboptimal relapse detection. Unsupervised clustering algorithms risked forming clusters based on sleep status rather than relapse status, due to the larger variance between sleep and awake data than between relapse and non-relapse states. This stressed the importance of stratifying by sleep status, especially in unsupervised learning tasks. We also observed person-specific behavioral patterns, with distinct feature distributions across individuals, supporting the value of personalized prediction. For example, User_01 had linear acceleration as a distinguishable feature (Fig. 3B) while User_00 did not (Fig. 3A).

To further assess the robustness of our algorithm, we repeated training and clustering under the best-performing configuration (Experiment 7: sleep data for both CAE training and clustering) using five different random seeds for initialization of CAE training and bootstrapping of test data for evaluation. Uncertainty measurements were reported in Table 4. The CAE achieved a reconstruction mean squared error (MSE) ranging from 0.043 to 0.088 across patients. Given that input features were normalized (mean = 0, standard deviation = 1), this corresponds to an average per-element error between approximately 0.21 and 0.29. These results indicate that the autoencoder effectively captured key temporal patterns in the 2D profiles, keeping relatively low reconstruction loss with small variability across seeds. Model performance on bootstrapped test data showed moderate variability, with standard deviations ranging from 0.012 to 0.138 and most around 0.05. Higher variability in evaluation metrics, including PR-AUC, ROC-AUC, and their harmonic mean, was generally associated with greater variability in reconstruction error. For example, User_08 exhibited a higher MSE standard deviation (0.055 ± 0.013), alongside increased uncertainty in PR-AUC (0.541 ± 0.106), ROC-AUC (0.656 ± 0.118), and harmonic mean (0.592 ± 0.138). Overall, these results demonstrate the stability of our approach, addressing variability from both model initialization and data sampling.

**Table 4:** Results for uncertainty analysis based on the best-performing experimental setup across five random seeds.

**Participant**	**MSE:** **Model initialization** **(mean **$\pm$** SD)**	**PR-AUC:** **Bootstrapping** **(mean**$\pm$**SD)**	**ROC-AUC:** **Bootstrapping** **(mean **$\pm$** SD)**	**Harmonic mean:** **Bootstrapping** **(mean **$\pm$** SD)**	**Baseline for PR-AUC** **i.e., positive rate**
User_00	0.047$\pm$0.005	0.637$\pm$0.036	0.739$\pm$0.091	0.682$\pm$0.058	0.244
User_01	0.067$\pm$0.004	0.863$\pm$0.014	0.600$\pm$0.117	0.702$\pm$0.085	0.713
User_02	0.068$\pm$0.013	0.637$\pm$0.074	0.661$\pm$0.157	0.641$\pm$0.095	0.333
User_03	0.077$\pm$0.005	0.727$\pm$0.020	0.678$\pm$0.070	0.700$\pm$0.044	0.447
User_04	0.043$\pm$0.004	0.539$\pm$0.054	0.664$\pm$0.086	0.591$\pm$0.054	0.148
User_05	0.088$\pm$0.008	0.733$\pm$0.032	0.641$\pm$0.051	0.682$\pm$0.032	0.440
User_06	0.056$\pm$0.004	0.604$\pm$0.030	0.640$\pm$0.133	0.614$\pm$0.069	0.156
User_07	0.063$\pm$0.011	0.800$\pm$0.018	0.569$\pm$0.026	0.665$\pm$0.019	0.764
User_08	0.055$\pm$0.013	0.541$\pm$0.106	0.656$\pm$0.118	0.592$\pm$0.138	0.222
User_09	0.064$\pm$0.007	0.881$\pm$0.012	0.596$\pm$0.062	0.709$\pm$0.047	0.771

## Discussion

This study presented a novel application of personalized relapse prediction for patients with psychotic disorders using objective measures derived from long-term, granular, and complete wearable data (such as physical and cardiac activities) via unsupervised anomaly detection. We implemented the algorithm using CAE and clustering methods to distinguish abnormal relapse-related patterns. We found potential systematic differences in relapse-related behavioral patterns between data collected during sleep and awake periods. By incorporating sleeping status in our analysis, e.g., training CAE and clustering relapse using data collected during sleep, we achieved promising results in experiments based on personalized schemes, comparable to the 1^st^ place on the SPGC leaderboard. Our results added to existing evidence that anomalies can be detected better using data collected during sleep periods^25^. Since most studies of relapse prediction for psychotic disorders based on other datasets focused on using less granular data (e.g., hourly, daily) and subjective measures (e.g., self-reported conditions), our study also filled the gap of personalized prediction relying on objective measures from nonobstructive wearables.

This work has several limitations, but it nonetheless highlights valuable future directions. First, the dataset only contains physiological signals from 10 patients with heterogeneous psychotic disorders. Further experiments are needed using data from more patients to ensure the robustness of our method. Second, the dataset defines relapse in a general clinical sense, without distinguishing between psychotic and non-psychotic relapses. The study sample includes a high proportion of patients with major mood episodes (e.g., bipolar disorder, schizoaffective disorder), which may contribute to a broader range of relapse phenotypes, including mood-related episodes without psychotic symptoms. While this reflects the heterogeneity observed in real-world clinical populations, it may obscure symptom-specific patterns relevant to different diagnostic subgroups. Future work can explore relapse subtype modeling using newer datasets (e.g., the 2024 SPGC dataset), which explicitly differentiate between psychotic and non-psychotic relapses^35^. This may allow more granular and diagnosis-specific prediction models. Third, we only used the features found in the SPGC baseline model, such as activities and heart rate variability metrics. Other objective measures related to physical activities, including steps and calories, can be considered^25^. Demographic (e.g., age, gender), socioeconomic, and environmental factors (e.g., social environments)^36,37^ can be also useful; however, this dataset does not include any of those. Moreover, the lack of information on medication use and treatment changes in the dataset can be another limitation since such factors can influence physiological signals and relapse dynamics. Finally, the dataset lacks labels for potential interactions (e.g., sleeping problems, lifestyle change)^38^. Despite our investigation into the stratification of sleeping status, future research can explore wearable-derived proxies of other life events and perform sophisticated stratification to ensure even less biased feature representation.

Most current studies of personalized, longitudinal relapse prediction in psychosis face the challenge of limited patient numbers (e.g., 5 to 20 relapse patients) due to the rarity of relapse events and difficulty in data collection^3,20,22^. At an early stage, this stresses the importance of collecting high-quality, long-term, consistent data — a focus increasingly embraced by various platforms, initiatives, and research teams^3,8,20,21,26,27^.

Our study offers new insights into mental health and digital phenotyping through unsupervised anomaly detection, exploring moment-by-moment individual-level data collected from personal digital devices. We contribute to the growing evidence base which supports the promising future of personalized preventive care for mental health conditions. Our early exploration shows great potential for relapse prediction to improve the life-quality of patients with psychotic disorders through remote monitoring and digital phenotyping, in the future.

## Methods

### Dataset

During the e-Prevention project, a total of 60 people were recruited, including 37 patients diagnosed with psychotic disorders and 23 healthy participants^3^. Among the patients, 15 withdrew during the assessment phase due to reasons unrelated to the study. Of the remaining 24 patients, only 10 had sufficient and consistent data for further analysis, after preprocessing the raw data and accounting for irrecoverable missing data. The e-Prevention SPGC released a public, de-identified dataset containing physiological signals of 10 patients with psychotic disorders, recorded in Samsung Gear S3 smartwatches over 2.5 years with a total of 2,699 days (relapse: 560, non-relapse: 2139). High-frequency physiological signals collected for each day included users’ linear and angular accelerations (20 Hz), heart rate and RR (R-peak-to-R-peak) intervals (5 Hz), sleep, and steps. Clinicians annotated daily relapse status by reviewing patients’ hospital assessments and communication with their physicians or family members.

The study which provided the above data was approved by the University Mental Health, Neurosciences and Precision Medicine Research Institute “Costas Stefanis’’ (UMHRI) in Athens, Greece. The data used in our study come from patients in the e-Prevention SPGC challenge that provided written informed consent and permission for use of their anonymized data for research. All analyses in the present study were conducted in accordance with the protocol approved for SPGC. We have shared synthetic data along with our code repository and recommend data requests be made directly to the original source (see Data Availability).

Data were split into training (only non-relapse), validation (both states with each labeled relapse and non-relapse), and test (both but unlabeled) sets. The non-relapse data were split approximately 8:1:1 into train, validation, and test sets, while the relapse data were divided 1:1 for validation and test sets. Splits were performed on a per-patient, per-event basis, as detailed in Table 1.

### Feature extraction, statistical analysis, normalization

For data preprocessing, we first removed outliers based on valid value ranges of accelerations, heart rate, and RR intervals^3^. Following the imputation techniques provided by e-prevention, in cases of missing data for up to 3 h, we imputed values using forward and backward linear interpolation^3,34^; when more than 3 consecutive hours of data were missing, we disregarded the entire interval. We removed noise in RR intervals’ ectopic peaks to generate NN (normal-to-normal) interval using NeuroKit^39^. Following preprocessing, we extracted 12 features on each non-overlapping 5-min interval, including the mean norm of linear and angular accelerations, the mean, maximum, and minimum of heart rate (beats per minute), the mean, standard deviation (SDNN), and root mean square of successive differences (RMSSD) of NN interval, low-frequency (LF) and high-frequency (HF) bands of NN interval, and major axis in the Poincare recurrence plot (standard deviations, SD1 and SD2).

We summarized the daily average of 5-min intervals and reported the mean and standard deviation for each feature distribution. For statistical analysis of the features, we applied the Kolmogorov–Smirnov (KS) test, a non-parametric test to determine the difference between two distributions, e.g., relapse vs. non-relapse^40^. KS statistics and adjusted p-values were reported to evaluate the level and significance of difference, respectively. We used the Benjamini–Hochberg procedure to adjust p-values for multiple comparisons^41^.

For data normalization, we computed each feature’s mean and standard deviation in the training set for each patient. Normalized training, validation, and test sets were based on the calculated mean and standard deviation per patient.

### Anomaly detection on personalized scheme

We adopted the steps of the unsupervised anomaly-detection framework summarized by Sunny et al., including data preprocessing, imputation, and unsupervised anomaly pattern extraction^33^. The occurrence of relapse and non-relapse data in the training and validation sets showed a large imbalance (Fig. 2A), so relapse was considered an anomaly and satisfied the assumption of such unsupervised methodology.

We trained models using a personalized scheme (Fig. 2B) where training and validation were performed for each patient separately for several reasons. First, the data were collected from various patients with different disorders. Second, the e-Prevention project did not achieve optimal model performance via global-scheme training (i.e., training performed for combined data) according to the low PR-AUC (median = 0.52), the area under the curve of the receiver operating characteristics (ROC-AUC) scores (median = 0.53)^3^. Most importantly, personalized predictions were particularly well-suited given person-dependent behavioral changes, aligning with the goal of patient-specific preventive care facilitated by the advancement of personal digital devices (Fig. 2C). We expected personalized models to be inherently generalizable as they relied on users’ own data for prediction.

### Learning latent representations of features via CAE

A total of 12 features extracted from the original data were stacked as 2D multivariable time-series profiles with a height of 48 and a width of 12. Each row represents a 5-min interval and each column represents feature, thus covering information from a consecutive 4-h window (Fig. 1).

We implemented a 2D CAE that learned to reconstruct 2D profiles using TensorFlow 2.2. The proposed CAE followed an encoder-decoder scheme, with one down-sampling block to map the input to a latent feature vector and an up-sampling block to reconstruct the input. The loss function was the MSE between the original and reconstructed 2D profiles. One down-sampling block consisted of a 2D convolutional layer (kernel size = 11, covering the whole 4-h window with a dimension of 12 rows) and a LeakyReLU activation. We used 15 latent features and 64 filters for the convolutional layer because we observed no significant decrease in MSE by varying these two parameters. We used a learning rate of 0.0001, employed by the SPGC baseline model. Early stopping was applied to monitor overfitting by stopping optimization after no decrease in validation loss after 3 epochs.

### Clustering relapse based on sleeping status using latent features

We then applied hard and soft clustering (e.g., k-means and Gaussian mixture models (GMM), respectively) using latent features as input (Fig. 1). K-means assumes well-separated clusters with one assigned cluster membership for each data point, while GMM generates probabilistic labels for data. During training, our algorithm first constructed latent features by optimizing CAE and then selected the optimal clustering method based on validation results (i.e., the harmonic mean score of PR-AUC and ROC-AUC of the validation set). We applied clustering as an unsupervised method, with the number of clusters preset to two to explore potential binary stratification patterns regarding relapse status within the data. The clustering algorithm (e.g., k-means or GMM) assigned cluster memberships without using any outcome labels. To evaluate the separation between clusters, we computed the silhouette score.

### Performance evaluation

Our algorithm generated relapse prediction for every 4-h window. Since evaluation occurred on a per-day basis, daily relapse scores were computed based on an unweighted average of all 4-h predictions during the day. We generated and reported PR-AUC and ROC-AUC of all 10 patients’ test data for model evaluation. We also reported per-patient evaluation results to show the robustness of our algorithm across different patients. Below, we presented how daily relapse score, aggregated evaluation metrics, and per-patient evaluation metrics were generated with Eq. (1), (2), and (3), respectively.

1 $$\widehat{Y} = \frac{\sum_{n=1}^{N}\widehat{{y}_{n}}}{N}$$

where $\widehat{Y}$ represents the predicted daily relapse score, N represents the total number of 4-h windows of the day, and $\widehat{{y}_{n}}$ represents the prediction of relapse status of the n-th 4-h window of the day.

2 $$AUC_{{aggregated}} = f\left( {all~predicted~events~from~all~patients} \right)$$

3 $${AUC}_{per-patient}=f(all~predicted~events~from~one~specified~patient)$$

where $f(\cdot )$ indicates an AUC function, e.g., PR-AUC, ROC-AUC. We computed the aggregated performance metric by pooling all predicted events and their corresponding true labels across all patients, and applying the evaluation function $f\left(\cdot \right)$.

### Uncertainty evaluation

To assess the stability and robustness of our approach, we repeated the training and clustering procedures under the best-performing configuration using five different random seeds. These seeds were used for both the initialization of CAE and the bootstrapping of the test data. We reported the uncertainty measurements, i.e., mean and standard deviation of the MSE for CAE training. We evaluated model performance on the bootstrapped test data by reporting the uncertainty of evaluation metrics including PR-AUC, ROC-AUC, and their harmonic mean.

## Supplementary Information

Supplementary Information.

## Data Availability

The public data underlying this article are available in Internet Archive Wayback Machine at https://robotics.ntua.gr/eprevention-sp-challenge/. You may contact the data owners listed on the webpage to get access to data.

## Code availability

Code and processed synthetic data generated for the analysis are available at https://doi.org/10.7281/T1XHQSBW.
