# Supplementary material for: Relapse prediction using wearable data through convolutional autoencoders and clustering for patients with psychotic disorders
Source: Sci Rep. 2025 May 29;15:18806. doi: 10.1038/s41598-025-03856-1 (PMC12122716; doi:10.1038/s41598-025-03856-1)
Supplement: Supplementary file 1 — Supplementary Information. [file 41598_2025_3856_MOESM1_ESM.docx]

**Supplementary Table 1. Patient-Specific Evaluation: Separate CAE Training and Clustering for Sleep/Awake**

| **Participant**  **(# day for evaluation)** | **Baseline for PR-AUC**  **(positive rate)** | **PR-AUC** | **ROC-AUC** | **Harmonic Mean** |
| --- | --- | --- | --- | --- |
| User_00 (41) | 0.244 | 0.643 | 0.760 | 0.697 |
| User_01 (80) | 0.713 | 0.875 | 0.583 | 0.700 |
| User_02 (39) | 0.333 | 0.705 | 0.672 | 0.688 |
| User_03 (38) | 0.447 | 0.727 | 0.528 | 0.612 |
| User_04 (27) | 0.148 | 0.585 | 0.564 | 0.575 |
| User_05 (50) | 0.440 | 0.673 | 0.514 | 0.583 |
| User_06 (32) | 0.156 | 0.585 | 0.602 | 0.593 |
| User_07 (123) | 0.764 | 0.788 | 0.505 | 0.615 |
| User_08 (18) | 0.222 | 0.468 | 0.531 | 0.498 |
| User_09 (96) | 0.771 | 0.883 | 0.543 | 0.673 |
| Median | - | **0.689** | 0.554 | 0.614 |
| SPGC Baseline | - | 0.635 | 0.578 | 0.605 |
| SPGC: PeRCeiVe | - | 0.651 | **0.647** | **0.649** |
| SPGC: Emotion | - | 0.635 | 0.607 | 0.621 |
| SPCG: SAILers | - | 0.636 | 0.584 | 0.605 |

**Supplementary Table 2. Patient-Specific Evaluation: CAE Training and Clustering Based on Sleep Data Only**

| **Participant (# day)**  **(# day for evaluation)** | **Baseline for PR-AUC**  **(positive rate)** | **PR-AUC** | **ROC-AUC** | **Harmonic Mean** |
| --- | --- | --- | --- | --- |
| User_00 (41) | 0.244 | 0.682 | 0.776 | 0.726 |
| User_01 (80) | 0.713 | 0.867 | 0.595 | 0.706 |
| User_02 (39) | 0.333 | 0.706 | 0.659 | 0.682 |
| User_03 (38) | 0.447 | 0.729 | 0.623 | 0.672 |
| User_04 (27) | 0.148 | 0.568 | 0.595 | 0.581 |
| User_05 (50) | 0.440 | 0.728 | 0.580 | 0.646 |
| User_06 (32) | 0.156 | 0.511 | 0.581 | 0.544 |
| User_07 (123) | 0.764 | 0.815 | 0.565 | 0.667 |
| User_08 (18) | 0.222 | 0.400 | 0.625 | 0.488 |
| User_09 (96) | 0.771 | 0.890 | 0.653 | 0.753 |
| Median | - | **0.717** | 0.609 | **0.670** |
| SPGC Baseline | - | 0.635 | 0.578 | 0.605 |
| SPGC: PeRCeiVe | - | 0.651 | **0.647** | 0.649 |
| SPGC: Emotion | - | 0.635 | 0.607 | 0.621 |
| SPCG: SAILers | - | 0.636 | 0.584 | 0.605 |

**Supplementary Table 3. Patient-Specific Evaluation: CAE Training and Clustering Based on Awake Data Only**

| **Participant (# day)**  **(# day for evaluation)** | **Baseline for PR-AUC**  **(positive rate)** | **PR-AUC** | **ROC-AUC** | **Harmonic Mean** |
| --- | --- | --- | --- | --- |
| User_00 (41) | 0.244 | 0.460 | 0.592 | 0.518 |
| User_01 (80) | 0.713 | 0.849 | 0.575 | 0.686 |
| User_02 (39) | 0.333 | 0.696 | 0.607 | 0.648 |
| User_03 (38) | 0.447 | 0.540 | 0.503 | 0.521 |
| User_04 (27) | 0.148 | 0.526 | 0.494 | 0.509 |
| User_05 (50) | 0.440 | 0.400 | 0.444 | 0.421 |
| User_06 (32) | 0.156 | 0.596 | 0.556 | 0.576 |
| User_07 (123) | 0.764 | 0.722 | 0.546 | 0.622 |
| User_08 (18) | 0.222 | 0.500 | 0.598 | 0.545 |
| User_09 (96) | 0.771 | 0.842 | 0.540 | 0.658 |
| Median | - | 0.568 | 0.551 | 0.561 |
| SPGC Baseline | - | 0.635 | 0.578 | 0.605 |
| SPGC: PeRCeiVe | - | **0.651** | **0.647** | **0.649** |
| SPGC: Emotion | - | 0.635 | 0.607 | 0.621 |
| SPCG: SAILers | - | 0.636 | 0.584 | 0.605 |

**Supplementary Table 4. Comparison of Feature Distributions Between Non-Relapse and Relapse Groups Stratified by Sleep Status.** The Kolmogorov-Smirnov (KS) test was performed.

|  |  | **Sleep** | | | | **Awake** | | | |
| --- | --- | --- | --- | --- | --- | --- | --- | --- | --- |
|  |  | **Non-relapse**  **(mean ± SD)** | **Relapse**  **(mean ± SD)** | **KS-statistic** | **p-value** | **Non-relapse**  **(mean ± SD)** | **Relapse**  **(mean ± SD)** | **KS-statistic** | **p-value** |
| **User_00** | Linear Acceleration (m/s^2^) | 0.22 ± 0.10 | 0.30 ± 0.04 | 0.68 | < 0.01* | 0.50 ± 0.17 | 0.68 ± 0.13 | 0.52 | 0.02* |
|  | Angular Acceleration (deg/s^2^) | 1.19 ± 1.36 | 0.57 ± 0.29 | 0.42 | 0.14 | 5.87 ± 2.52 | 7.96 ± 2.74 | 0.36 | 0.17 |
|  | Heart Rate (beats-per-minute) | 81 ± 14 | 89 ± 17 | 0.75 | < 0.01* | 91 ± 14 | 102 ± 10 | 0.57 | < 0.01* |
|  | RR Interval (ms) | 759 ± 89 | 683 ± 17 | 0.79 | < 0.01* | 697 ± 60 | 681 ± 16 | 0.39 | 0.12 |
|  | RMSSD (ms) | 65.6 ± 25.0 | 32.5 ± 9.0 | 0.87 | < 0.01* | 126.4 ± 38.4 | 124.6 ± 15.6 | 0.5 | 0.02* |
|  | SDNN (ms) | 56.5 ± 20.7 | 27.2 ± 7.6 | 0.9 | < 0.01* | 110.4 ± 34.3 | 99.1 ± 10.4 | 0.6 | < 0.01* |
|  | Poincare major axis SD1 | 46.3 ± 17.7 | 23.0 ± 6.3 | 0.87 | < 0.01* | 89.2 ± 27.3 | 88.1 ± 11.0 | 0.5 | 0.02* |
|  | Poincare major axis SD2 | 63.0 ± 23.7 | 30.0 ± 8.5 | 0.9 | < 0.01* | 124.0 ± 41.0 | 106.5 ± 10.9 | 0.61 | < 0.01* |
|  | LF band (Hz) | 0.02 ± 0.03 | 0.01 ± 0.00 | 0.36 | < 0.01* | 0.02 ± 0.03 | 0.03 ± 0.01 | 0.51 | < 0.01* |
|  | HF band (Hz) | 0.03 ± 0.06 | 0.03 ± 0.01 | 0.67 | 0.28 | 0.04 ± 0.06 | 0.06 ± 0.03 | 0.64 | 0.02* |
|  | | | | | | | | | |
| **User_01** | Linear Acceleration (m/s^2^) | 0.14 ± 0.04 | 0.18 ± 0.04 | 0.45 | < 0.01* | 0.66 ± 0.18 | 0.66 ± 0.17 | 0.16 | 0.4 |
|  | Angular Acceleration (deg/s^2^) | 0.58 ± 0.45 | 0.55 ± 0.53 | 0.14 | 0.46 | 8.20 ± 2.54 | 7.71 ± 2.37 | 0.22 | 0.25 |
|  | Heart Rate (beats-per-minute) | 63 ± 8 | 66 ± 11 | 0.16 | 0.33 | 92 ± 13 | 90 ± 14 | 0.2 | 0.4 |
|  | RR Interval (ms) | 990 ± 90 | 956 ± 100 | 0.25 | < 0.01* | 785 ± 55 | 761 ± 79 | 0.23 | 0.02* |
|  | RMSSD (ms) | 57.8 ± 17.8 | 46.8 ± 12.4 | 0.41 | < 0.01* | 138.1 ± 26.8 | 124.7 ± 28.6 | 0.3 | < 0.01* |
|  | SDNN (ms) | 51.8 ± 13.8 | 43.9 ± 12.7 | 0.36 | < 0.01* | 112.5 ± 20.6 | 100.6 ± 22.7 | 0.31 | < 0.01* |
|  | Poincare major axis SD1 | 40.9 ± 12.6 | 33.1 ± 8.8 | 0.41 | < 0.01* | 97.6 ± 18.9 | 88.2 ± 20.3 | 0.3 | < 0.01* |
|  | Poincare major axis SD2 | 59.3 ± 15.0 | 51.1 ± 14.7 | 0.3 | < 0.01* | 122.6 ± 22.0 | 108.9 ± 24.6 | 0.32 | < 0.01* |
|  | LF band (Hz) | 0.01 ± 0.00 | 0.01 ± 0.01 | 0.28 | < 0.01* | 0.02 ± 0.02 | 0.03 ± 0.03 | 0.19 | 0.03* |
|  | HF band (Hz) | 0.01 ± 0.01 | 0.02 ± 0.02 | 0.11 | 0.64 | 0.05 ± 0.04 | 0.07 ± 0.06 | 0.19 | 0.03* |
|  | | | | | | | | | |
| **User_02** | Linear Acceleration (m/s^2^) | 0.14 ± 0.05 | 0.14 ± 0.03 | 0.22 | 0.91 | 0.52 ± 0.21 | 0.54 ± 0.26 | 0.17 | 0.85 |
|  | Angular Acceleration (deg/s^2^) | 1.01 ± 0.77 | 0.80 ± 0.38 | 0.20 | 0.91 | 5.94 ± 2.46 | 6.27 ± 2.59 | 0.29 | 0.33 |
|  | Heart Rate (beats-per-minute) | 79 ± 23 | 69 ± 3 | 0.63 | 0.03* | 91 ± 16 | 88 ± 15 | 0.24 | 0.49 |
|  | RR Interval (ms) | 785 ± 107 | 863 ± 32 | 0.61 | 0.03* | 698 ± 72 | 738 ± 97 | 0.35 | 0.33 |
|  | RMSSD (ms) | 44.4 ± 24.8 | 38.7 ± 4.7 | 0.37 | 0.33 | 104.1 ± 30.8 | 112.0 ± 25.9 | 0.3 | 0.33 |
|  | SDNN (ms) | 42.2 ± 24.2 | 34.1 ± 3.9 | 0.42 | 0.27 | 93.2 ± 28.1 | 99.5 ± 22.4 | 0.3 | 0.33 |
|  | Poincare major axis SD1 | 31.4 ± 17.5 | 27.4 ± 3.3 | 0.37 | 0.33 | 73.4 ± 21.9 | 79.2 ± 18.3 | 0.3 | 0.33 |
|  | Poincare major axis SD2 | 48.9 ± 29.1 | 38.6 ± 4.5 | 0.42 | 0.27 | 105.3 ± 34.7 | 112.6 ± 25.9 | 0.35 | 0.33 |
|  | LF band (Hz) | 0.04 ± 0.03 | 0.02 ± 0.01 | 0.38 | 0.05* | 0.03 ± 0.03 | 0.02 ± 0.01 | 0.28 | 0.33 |
|  | HF band (Hz) | 0.04 ± 0.06 | 0.02 ± 0.01 | 0.56 | 0.33 | 0.05 ± 0.06 | 0.03 ± 0.02 | 0.35 | 0.33 |
|  | | | | | | | | | |
| **User_03** | Linear Acceleration (m/s^2^) | 0.25 ± 0.04 | 0.29 ± 0.08 | 0.43 | 0.13 | 0.67 ± 0.22 | 0.67 ± 0.19 | 0.17 | 0.78 |
|  | Angular Acceleration (deg/s^2^) | 0.82 ± 0.37 | 1.18 ± 1.08 | 0.33 | 0.24 | 5.42 ± 1.49 | 5.84 ± 2.20 | 0.22 | 0.62 |
|  | Heart Rate (beats-per-minute) | 76 ± 10 | 78 ± 8 | 0.27 | 0.42 | 94 ± 6 | 97 ± 13 | 0.3 | 0.37 |
|  | RR Interval (ms) | 858 ± 42 | 871 ± 48 | 0.31 | 0.27 | 730 ± 34 | 717 ± 58 | 0.49 | < 0.01* |
|  | RMSSD (ms) | 50.0 ± 9.9 | 63.1 ± 21.0 | 0.38 | 0.13 | 117.7 ± 20.7 | 112.2 ± 26.4 | 0.28 | 0.37 |
|  | SDNN (ms) | 46.8 ± 8.6 | 56.7 ± 15.6 | 0.38 | 0.13 | 99.0 ± 16.9 | 96.1 ± 24.0 | 0.19 | 0.78 |
|  | Poincare major axis SD1 | 35.3 ± 7.0 | 44.6 ± 14.8 | 0.38 | 0.13 | 83.2 ± 14.6 | 79.3 ± 18.6 | 0.28 | 0.37 |
|  | Poincare major axis SD2 | 54.6 ± 9.8 | 64.7 ± 16.1 | 0.38 | 0.13 | 109.7 ± 18.9 | 107.3 ± 28.2 | 0.18 | 0.78 |
|  | LF band (Hz) | 0.02 ± 0.01 | 0.01 ± 0.01 | 0.25 | 0.64 | 0.02 ± 0.01 | 0.02 ± 0.02 | 0.14 | 0.87 |
|  | HF band (Hz) | 0.03 ± 0.03 | 0.02 ± 0.01 | 0.21 | 0.48 | 0.04 ± 0.03 | 0.04 ± 0.04 | 0.25 | 0.51 |
|  | | | | | | | | | |
| **User_04** | Linear Acceleration (m/s^2^) | 0.17 ± 0.05 | 0.17 ± 0.09 | 0.44 | 0.86 | 0.55 ± 0.11 | 0.36 ± 0.02 | 0.95 | 0.03* |
|  | Angular Acceleration (deg/s^2^) | 0.40 ± 0.21 | 0.38 ± 0.09 | 0.33 | 0.96 | 6.64 ± 1.64 | 4.15 ± 0.45 | 0.91 | 0.03* |
|  | Heart Rate (beats-per-minute) | 62 ± 10 | 56 ± 8 | 0.49 | 0.86 | 86 ± 12 | 77 ± 9 | 0.62 | 0.33 |
|  | RR Interval (ms) | 1015 ± 96 | 1140 ± 87 | 0.77 | 0.23 | 817 ± 62 | 803 ± 114 | 0.46 | 0.66 |
|  | RMSSD (ms) | 67.9 ± 16.7 | 86.6 ± 9.6 | 0.83 | 0.16 | 132.7 ± 22.6 | 97.2 ± 4.5 | 0.93 | 0.03* |
|  | SDNN (ms) | 60.8 ± 15.0 | 81.1 ± 2.4 | 0.94 | 0.04* | 110.4 ± 17.8 | 84.4 ± 5.4 | 0.9 | 0.03* |
|  | Poincare major axis SD1 | 48.0 ± 11.8 | 61.2 ± 6.8 | 0.83 | 0.16 | 93.6 ± 16.0 | 68.7 ± 3.2 | 0.93 | 0.03* |
|  | Poincare major axis SD2 | 69.3 ± 17.3 | 93.8 ± 0.7 | 0.95 | 0.04* | 121.8 ± 19.8 | 94.8 ± 6.7 | 0.87 | 0.05* |
|  | LF band (Hz) | 0.01 ± 0.01 | 0.01 ± 0.00 | 0.46 | 0.86 | 0.02 ± 0.02 | 0.05 ± 0.02 | 0.91 | 0.03* |
|  | HF band (Hz) | 0.02 ± 0.03 | 0.01 ± 0.00 | 0.42 | 0.86 | 0.04 ± 0.04 | 0.05 ± 0.02 | 0.9 | 0.03* |
|  | | | | | | | | | |
| **User_05** | Linear Acceleration (m/s^2^) | 0.17 ± 0.05 | 0.17 ± 0.09 | 0.44 | 0.86 | 0.48 ± 0.21 | 0.46 ± 0.15 | 0.16 | 0.68 |
|  | Angular Acceleration (deg/s^2^) | 0.4 ± 0.21 | 0.38 ± 0.09 | 0.33 | 0.96 | 4.18 ± 1.81 | 4.79 ± 2.00 | 0.26 | 0.32 |
|  | Heart Rate (beats-per-minute) | 62 ± 10 | 56 ± 8 | 0.49 | 0.86 | 78 ± 8 | 89 ± 8 | 0.6 | <0.01* |
|  | RR Interval (ms) | 1015 ± 96 | 1140 ± 87 | 0.77 | 0.23 | 866 ± 82 | 752 ± 67 | 0.64 | <0.01* |
|  | RMSSD (ms) | 67.9 ± 16.7 | 86.6 ± 9.6 | 0.83 | 0.16 | 110.6 ± 24.9 | 107.8 ± 29.3 | 0.21 | 0.43 |
|  | SDNN (ms) | 60.8 ± 15.0 | 81.1 ± 2.4 | 0.94 | 0.04* | 96.9 ± 21.0 | 90.3 ± 23.1 | 0.23 | 0.43 |
|  | Poincare major axis SD1 | 48.0 ± 11.8 | 61.2 ± 6.8 | 0.83 | 0.16 | 78.2 ± 17.6 | 76.2 ± 20.7 | 0.21 | 0.43 |
|  | Poincare major axis SD2 | 69.3 ± 17.3 | 93.8 ± 0.7 | 0.95 | 0.04* | 109.5 ± 23.7 | 99.8 ± 25.1 | 0.26 | 0.32 |
|  | LF band (Hz) | 0.01 ± 0.01 | 0.01 ± 0.00 | 0.46 | 0.86 | 0.01 ± 0.01 | 0.01 ± 0.01 | 0.2 | 0.43 |
|  | HF band (Hz) | 0.02 ± 0.03 | 0.01 ± 0.00 | 0.42 | 0.86 | 0.02 ± 0.02 | 0.02 ± 0.02 | 0.16 | 0.68 |
|  | | | | | | | | | |
| **User_06** | Linear Acceleration (m/s^2^) | 0.19 ± 0.05 | 0.15 ± 0.00 | 0.82 | 0.61 | 0.42 ± 0.15 | 0.36 ± 0.01 | 0.61 | 0.52 |
|  | Angular Acceleration (deg/s^2^) | 0.88 ± 0.68 | 0.69 ± 0.20 | 0.32 | 0.97 | 4.40 ± 1.80 | 3.59 ± 0.12 | 0.68 | 0.52 |
|  | Heart Rate (beats-per-minute) | 64 ± 9 | 60 ± 2 | 0.54 | 0.61 | 82 ± 17 | 73 ± 4 | 0.73 | 0.52 |
|  | RR Interval (ms) | 974 ± 111 | 1008 ± 48 | 0.49 | 0.69 | 799 ± 97 | 876 ± 29 | 0.77 | 0.52 |
|  | RMSSD (ms) | 62.1 ± 21.3 | 64.2 ± 4.3 | 0.56 | 0.61 | 116.0 ± 33.9 | 113.8 ± 18.8 | 0.41 | 0.62 |
|  | SDNN (ms) | 61.2 ± 22.5 | 64.8 ± 5.6 | 0.58 | 0.61 | 98.6 ± 26.8 | 91.9 ± 12.5 | 0.51 | 0.8 |
|  | Poincare major axis SD1 | 43.9 ± 15.0 | 45.4 ± 3.0 | 0.56 | 0.61 | 82.0 ± 24.0 | 80.4 ± 13.3 | 0.41 | 0.62 |
|  | Poincare major axis SD2 | 72.6 ± 27.9 | 78.0 ± 7.5 | 0.6 | 0.61 | 109.7 ± 29.3 | 99.2 ± 12.0 | 0.56 | 0.56 |
|  | LF band (Hz) | 0.01 ± 0.02 | 0.01 ± 0.00 | 0.41 | 0.88 | 0.02 ± 0.03 | 0.01 ± 0.00 | 0.62 | 0.52 |
|  | HF band (Hz) | 0.02 ± 0.03 | 0.01 ± 0.00 | 0.56 | 0.61 | 0.05 ± 0.07 | 0.01 ± 0.00 | 0.72 | 0.52 |
|  | | | | | | | | | |
| **User_07** | Linear Acceleration (m/s^2^) | 0.30 ± 0.07 | 0.31 ± 0.05 | 0.17 | 0.06 | 0.54 ± 0.27 | 0.44 ± 0.07 | 0.20 | < 0.01* |
|  | Angular Acceleration (deg/s^2^) | 1.09 ± 1.21 | 1.16 ± 0.68 | 0.28 | < 0.01* | 6.15 ± 6.45 | 3.93 ± 1.25 | 0.18 | 0.02* |
|  | Heart Rate (beats-per-minute) | 64 ± 7 | 71 ± 6 | 0.49 | < 0.01* | 79 ± 8 | 82 ± 10 | 0.26 | < 0.01* |
|  | RR Interval (ms) | 957 ± 88 | 861 ± 71 | 0.53 | < 0.01* | 813 ± 66 | 769 ± 62 | 0.31 | < 0.01* |
|  | RMSSD (ms) | 48.0 ± 20.4 | 39.7 ± 11.3 | 0.27 | < 0.01* | 87.6 ± 20.6 | 78.4 ± 19.9 | 0.21 | < 0.01* |
|  | SDNN (ms) | 45.2 ± 16.5 | 36.7 ± 8.7 | 0.33 | < 0.01* | 76.2 ± 16.9 | 65.0 ± 16.6 | 0.30 | < 0.01* |
|  | Poincare major axis SD1 | 33.9 ± 14.4 | 28.0 ± 8.0 | 0.27 | < 0.01* | 61.9 ± 14.6 | 55.4 ± 14.1 | 0.21 | < 0.01* |
|  | Poincare major axis SD2 | 52.7 ± 18.3 | 42.4 ± 9.4 | 0.34 | < 0.01* | 85.7 ± 18.9 | 71.3 ± 18.6 | 0.36 | < 0.01* |
|  | LF band (Hz) | 0.01 ± 0.01 | 0.03 ± 0.01 | 0.62 | < 0.01* | 0.02 ± 0.02 | 0.02 ± 0.02 | 0.21 | < 0.01* |
|  | HF band (Hz) | 0.02 ± 0.02 | 0.02 ± 0.02 | 0.35 | < 0.01* | 0.03 ± 0.03 | 0.03 ± 0.05 | 0.43 | < 0.01* |
|  | | | | | | | | | |
| **User_08** | Linear Acceleration (m/s^2^) | 0.19 ± 0.14 | 0.72 | 1.0 | 0.03* | 0.37 ± 0.15 | 0.24 ± 0.00 | 0.78 | 0.53 |
|  | Angular Acceleration (deg/s^2^) | 1.94 ± 2.14 | 9.26 | 0.96 | 0.1 | 4.38 ± 2.09 | 2.51 ± 0.26 | 0.83 | 0.53 |
|  | Heart Rate (beats-per-minute) | 73 ± 27 | 96 | 0.89 | 0.24 | 77 ± 23 | 74 ± 6 | 0.52 | 0.80 |
|  | RR Interval (ms) | 864 ± 172 | 752 | 0.86 | 0.31 | 760 ± 130 | 738 ± 253 | 0.44 | 0.85 |
|  | RMSSD (ms) | 78.5 ± 34.9 | 153.6 | 0.96 | 0.1 | 101.1 ± 40.2 | 82.5 ± 30.6 | 0.6 | 0.80 |
|  | SDNN (ms) | 74.1 ± 37.6 | 124.5 | 0.89 | 0.24 | 89.0 ± 33.3 | 76.5 ± 37.3 | 0.4 | 0.85 |
|  | Poincare major axis SD1 | 55.5 ± 24.6 | 108.6 | 0.96 | 0.1 | 71.5 ± 28.4 | 58.3 ± 21.6 | 0.6 | 0.80 |
|  | Poincare major axis SD2 | 85.6 ± 47.4 | 136.6 | 0.89 | 0.24 | 100.2 ± 38.4 | 87.5 ± 46.7 | 0.39 | 0.85 |
|  | LF band (Hz) | 0.02 ± 0.04 | 0.01 | 1.0 | 0.35 | 0.04 ± 0.05 | 0.04 ± 0.05 | 0.53 | 0.80 |
|  | HF band (Hz) | 0.05 ± 0.09 | 0.01 | 0.84 | 0.03* | 0.08 ± 0.10 | 0.10 ± 0.11 | 0.43 | 0.85 |
|  | | | | | | | | | |
| **User_09** | Linear Acceleration (m/s^2^) | 0.14 ± 0.08 | 0.14 ± 0.07 | 0.1 | 0.80 | 0.52 ± 0.14 | 0.50 ± 0.12 | 0.16 | 0.12 |
|  | Angular Acceleration (deg/s^2^) | 1.41 ± 1.09 | 1.29 ± 0.83 | 0.16 | 0.32 | 7.78 ± 2.19 | 7.56 ± 1.93 | 0.17 | 0.12 |
|  | Heart Rate (beats-per-minute) | 82 ± 7 | 85 ± 6 | 0.28 | < 0.01* | 93 ± 8 | 94 ± 6 | 0.2 | 0.06 |
|  | RR Interval (ms) | 751 ± 57 | 717 ± 48 | 0.3 | < 0.01* | 716 ± 44 | 702 ± 34 | 0.2 | 0.06 |
|  | RMSSD (ms) | 35.6 ± 13.6 | 35.5 ± 9.6 | 0.13 | 0.50 | 107.5 ± 27.5 | 101.3 ± 25.9 | 0.23 | 0.03* |
|  | SDNN (ms) | 33.2 ± 10.1 | 32.2 ± 6.7 | 0.11 | 0.76 | 85.3 ± 21.2 | 80.0 ± 19.8 | 0.24 | 0.03* |
|  | Poincare major axis SD1 | 25.2 ± 9.6 | 25.1 ± 6.8 | 0.13 | 0.50 | 76.0 ± 19.4 | 71.6 ± 18.3 | 0.23 | 0.03* |
|  | Poincare major axis SD2 | 38.3 ± 10.7 | 36.7 ± 7.1 | 0.17 | 0.25 | 91.3 ± 22.5 | 85.6 ± 21.0 | 0.22 | 0.03* |
|  | LF band (Hz) | 0.03 ± 0.01 | 0.04 ± 0.02 | 0.13 | < 0.01* | 0.03 ± 0.03 | 0.03 ± 0.03 | 0.16 | 0.12 |
|  | HF band (Hz) | 0.01 ± 0.00 | 0.01 ± 0.00 | 0.27 | 0.56 | 0.06 ± 0.07 | 0.06 ± 0.06 | 0.22 | 0.03* |
|  | | | | | | | | | |
| **Combined** | Linear Acceleration (m/s^2^) | 0.20 ± 0.09 | 0.22 ± 0.10 | 0.21 | < 0.01 | 0.53 ± 0.21 | 0.52 ± 0.17 | 0.09 | 0.04 |
|  | Angular Acceleration (deg/s^2^) | 0.10 ± 1.03 | 1.05 ± 0.95 | 0.12 | < 0.01 | 5.91 ± 3.23 | 5.98 ± 2.55 | 0.10 | 0.02 |
|  | Heart Rate (beats-per-minute) | 71 ± 15 | 74 ± 11 | 0.19 | < 0.01 | 86 ± 14 | 89 ± 12 | 0.15 | < 0.01 |
|  | RR Interval (ms) | 900 ± 136 | 850 ± 118 | 0.23 | < 0.01 | 769 ± 91 | 743 ± 70 | 0.19 | < 0.01 |
|  | RMSSD (ms) | 58.5 ± 25.6 | 44.1 ± 18.2 | 0.36 | < 0.01 | 114.5 ± 32.4 | 100.7 ± 30.1 | 0.21 | < 0.01 |
|  | SDNN (ms) | 53.8 ± 22.9 | 40.4 ± 15.3 | 0.39 | < 0.01 | 97.6 ± 26.8 | 82.4 ± 24.2 | 0.26 | < 0.01 |
|  | Poincare major axis SD1 | 41.3 ± 18.1 | 31.2 ± 12.9 | 0.36 | < 0.01 | 80.9 ± 23.0 | 71.2 ± 21.3 | 0.21 | < 0.01 |
|  | Poincare major axis SD2 | 62.0 ± 26.8 | 46.5 ± 17.3 | 0.39 | < 0.01 | 108.5 ± 30.6 | 89.8 ± 26.7 | 0.29 | < 0.01 |
|  | LF band (Hz) | 0.02 ± 0.02 | 0.02 ± 0.02 | 0.11 | < 0.01 | 0.02 ± 0.02 | 0.03 ± 0.02 | 0.08 | < 0.01 |
|  | HF band (Hz) | 0.02 ± 0.04 | 0.02 ± 0.02 | 0.34 | < 0.01 | 0.04 ± 0.05 | 0.05 ± 0.06 | 0.26 | 0.1 |

Abbreviations: SD (standard deviation), KS (Kolmogorov-Smirnov), m/s^2^ (meter per second^2^), deg/s^2^ (degree per second^2^), ms (millisecond), Hz (Hertz), RR (R-peak-to-R-peak), RMSSD (root mean square of successive differences), SDNN (standard deviation of normal-to-normal interval), LF (low-frequency), HF (high-frequency)

We used the Benjamini-Hochberg procedure to adjust p-values for multiple comparisons. Significant p-values (p < 0.05) are indicated by *. The KS statistic ranges from 0 to 1, with lower values suggesting a higher likelihood that the two samples are drawn from the same distribution.


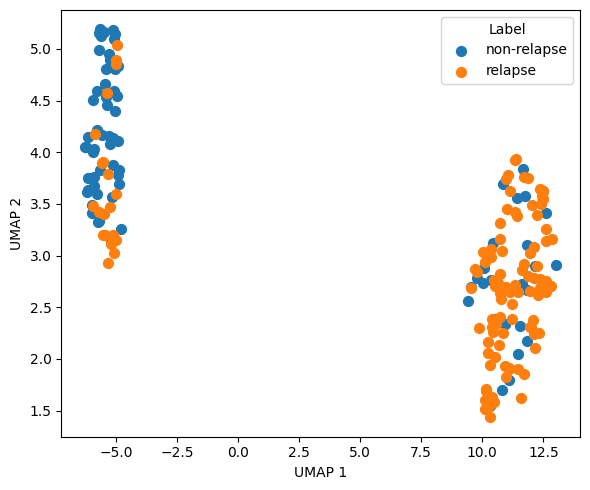


**Supplementary Figure 1. UMAP Projection of Latent Representations Colored by Ground-Truth Labels (Non-Relapse vs. Relapse)**
